# Elastic stable intramedullary nailing vs. hip spica casting management for postoperative femoral fractures in children with developmental dysplasia of the hip

**DOI:** 10.3389/fped.2026.1765117

**Published:** 2026-06-22

**Authors:** Yongfu Wang, Li Yang, Qiang Shi

**Affiliations:** Department of Spine Surgery Zone 1, The Affiliated Changsha Central Hospital, Hengyang Medical School, University of South China, Changsha, China

**Keywords:** children, DDH, elastic stable intramedullary nailing, femoral fracture, hip spica casting

## Abstract

**Introduction:**

Proximal femoral osteotomy (PFO) produces excellent clinical results for developmental dysplasia of the hip (DDH) in children. Femur fractures in the hips of children after PFO have been reported, and both elastic stable intramedullary nailing (ESIN) and hip spica casting have been used to treat these fractures. Usually, decision-making between ESIN and hip spica casting has been based on the fracture pattern and age. The purpose of this study was to compare the outcomes of elastic stable intramedullary nailing with those of hip spica casting management for postoperative femoral fractures in the hips of children with developmental dysplasia.

**Methods:**

Ten patients were classified into the ESIN group, whereas eight patients were classified into the hip spica casting group. The age, fracture pattern, average hospitalization, surgical time, and complications were compared between the two groups.

**Results:**

From July 2012 to July 2018, a total of 396 children with DDH were treated at our institution, and 18 patients who presented with postoperative femur fractures were included in the study. There were significant differences in age and fracture pattern (*P* < 0.001). Conversely, no statistically significant difference was found between the two groups concerning gender and side. Meanwhile, patients with a follow-up time of <24 months or incomplete medical records were excluded from our study. Compared with that in the ESIN (6.4 days), the average length of hospitalization was longer in the hip spica casting group (9.5 days, *P* < 0.001). The surgical time was shorter in the hip spica casting group (24.8 ± 4.5 min) than in the ESIN group (34.7 ± 5.1 min) (*P* < 0.05).

**Conclusion:**

Our study revealed that the radiological outcomes were generally good after ESIN or hip spica casting. In our study, hip spica casting was suggested for undisplaced femoral fractures in patients aged 2–5 years. In addition, ESIN was a quick and minimally invasive technique for treating displaced femoral fractures in patients aged 5–8 years and prevented secondary displacement.

## Background

Proximal femoral osteotomy (PFO), including femoral shortening, varus osteotomy, or derotation osteotomy, is usually performed to correct proximal femoral deformities in developmental dysplasia of the hip (DDH) (1). To maintain the stability of the osteotomy site, locking compression plates (LCPs) are being increasingly utilized in the pediatric population (2). However, the risk of femur fractures after osteotomy has been reported in recent years (3, 4). The time for implant removal after PFO in children with DDH is 12–18 months, which is typically when solid union is routinely observed. Although hip spica casting and elastic stable intramedullary nailing (ESIN) have been demonstrated to be effective for femur fractures (5, 6), the optimal postoperative treatment method for femur fractures in patients with DDH remains largely ambiguous.

The purpose of this study was to compare the outcomes of elastic stable intramedullary nailing to those of hip spica casting management for postoperative femoral fractures in the hips of children with developmental dysplasia.

## Materials and methods

### Patients

The Ethics Review Committee of Changsha Central Hospital approved our retrospective study, and all parents provided informed consent to participate. Generally, we decreased the anteversion angle to no less than 30°, decreased the neck–shaft angle to no less than 120°, and shortened the femur for complete dislocation. Moreover, capsulotomy was usually performed in children with Tönnis grade III/IV and sometimes in those with Tönnis grade II to enable reduction and capsulorrhaphy. The extent of dislocation of the femoral head, neck–shaft angle, and anteversion angle in the proximal femur were evaluated by preoperative radiography and computed tomography (CT). All patients underwent open reduction, femoral osteotomy, and pelvic osteotomy (Salter pelvic osteotomy for 15 patients and Pemberton pelvic osteotomy for 3 patients) in our hospital. The associated conditions of the patients included pain and motion limitations of the hip. Patients with metabolic bone disease, neuromuscular conditions, bone fragility disorders, nutritional deficiencies, and pathologic fractures were excluded from the present study. The fracture location and refixation method were recorded for all patients. Patients (*n* = 10) who underwent ESIN fixation surgery were classified into Group A, while those (*n* = 8) who underwent hip spica casting were classified into Group B.

The inclusion criteria were as follows: (a) postoperative femur fractures of the hip (the anatomy of the proximal femur, the implant and osteotomy site, and the distal femur); (b) fractures that occurred within 2 years after PFO, regardless of whether the plate was removed; and (c) the absence of a history of severe trauma, including a fall from standing, twisting, or walking. The average hospitalization, surgical time, and complications were compared between the two groups.

### Surgical procedures

In our hospital, Salter pelvic osteotomy and PFO were usually performed in children with DDH who were between 2 and 6 years old, and Pemberton pelvic osteotomy and PFO were usually performed in children with DDH who were between 6 and 8 years old. After PFO, LCPs were used for the fixation of osteotomies in the pediatric population. Moreover, implant removal after PFO in children with DDH was performed 12–18 months after solid union was routinely observed. If femur fractures of the hip occurred after the operation, ESIN or hip spica casting was performed. All surgeries in the children included in this study were performed by the same experienced pediatric orthopedic surgeon on a radiolucent fracture table.

**Figure 1:**
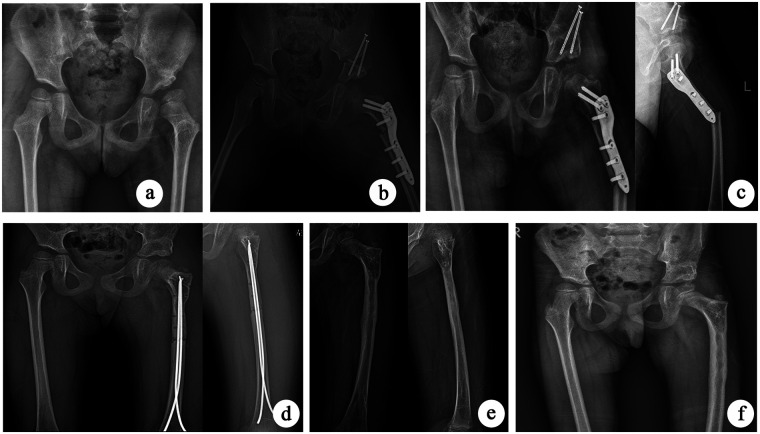
Left DDH in a 5-year-old girl. **(a)** Preoperative radiograph. **(b)** After Salter and proximal femoral osteotomy. **(c)** She had a femoral fracture in the screw hole at 9 months postoperatively when her femur was injured after a domestic fall. **(d)** ESIN was performed after removal of internal fixation. **(e)** Six-month radiograph after ESIN was removed. **(f)** Twelve-month radiograph after ESIN was removed. DDH, developmental dysplasia of the hip; ESIN, elastic stable intramedullary nailing.

In the ESIN group (Figure 1), ESIN (Synthes, Solothurn, Switzerland) was inserted after achieving fracture reduction by traction. The nail diameter was predetermined to fill the medullary canal with 35%–40% of the narrowest diameter of the femur. After 2–3 weeks, the children and their parents were instructed to properly move the adjacent joints and begin partial weight-bearing for approximately 4 weeks. When a bridging callus appeared and the fracture line was no longer visible on the X-ray film, partial weight-bearing progressed to full weight-bearing. The time for initiation of weight-bearing exercises was determined on the basis of the fracture type and the X-ray results during the follow-up period. The alignment and healing of the fractures were reviewed using radiographs at 1, 3, and 6 months postoperatively. The children were evaluated clinically and radiologically at each follow-up examination, and the complications were recorded. When the fracture was fully healed (3–6 months), the internal fixator was removed at approximately 6 months under general anesthesia in the operating room.

**Figure 2:**
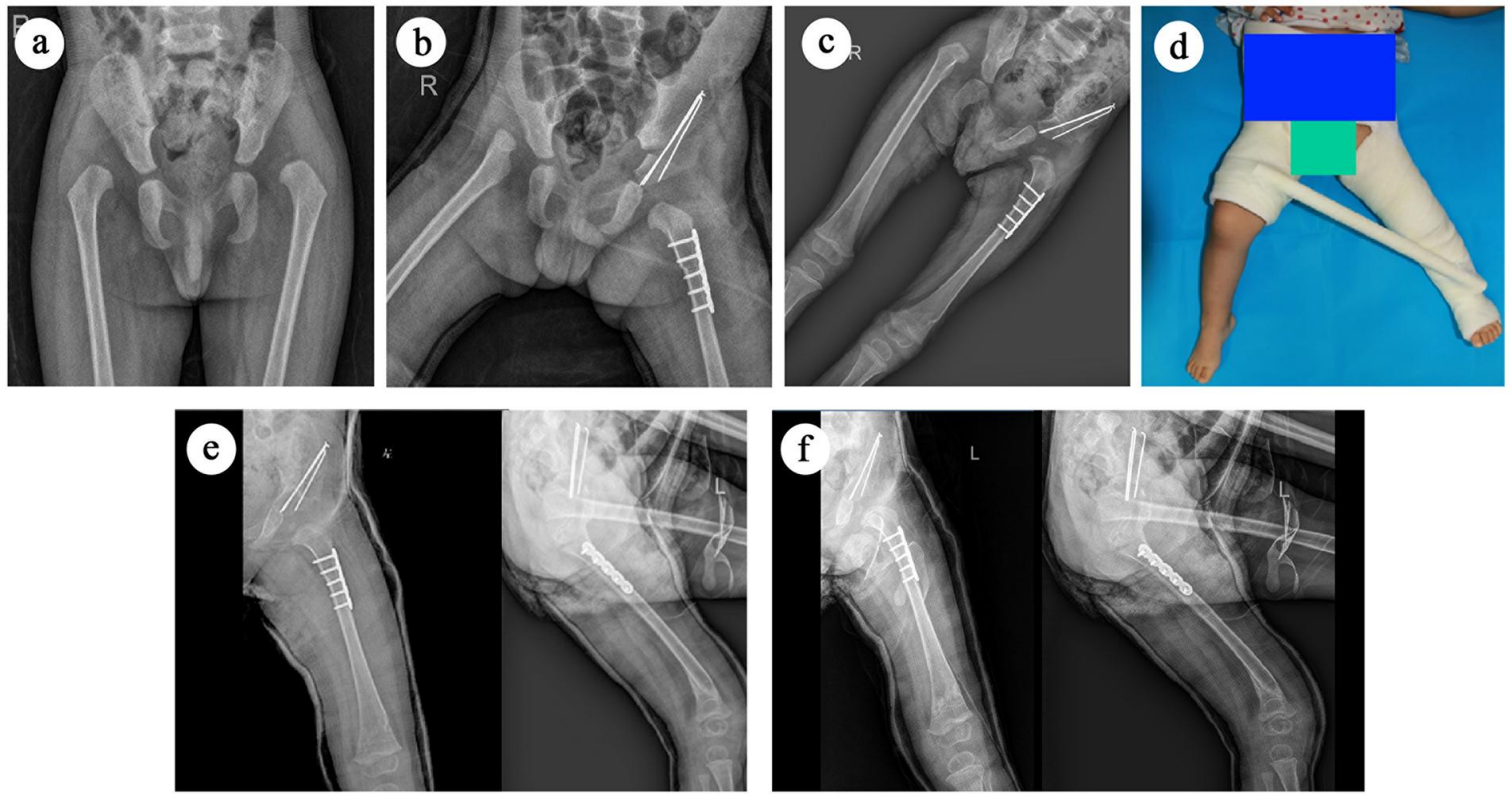
Bilateral DDH in a 2-year-old girl. **(a)** Radiograph showing bilateral DDH. **(b)** Salter and left proximal femoral osteotomy. **(c)** A mildly displaced fracture of the distal femur was found 1 month after PFO. **(d)** Appearance after early spica cast. **(e)** Radiograph obtained the day after early spica casting. **(f)** Two-month radiograph after spica cast fixation. DDH, developmental dysplasia of the hip; PFO, proximal femoral osteotomy.

In the spica casting group (Figure 2), children with femur fractures of the hip after the operation were treated with skin traction for 7 days, and then general anesthesia was administered to ensure successful hip spica casting with the aid of radiography in the operating theater. In our study, the hips were flexed up to 40°, abducted up to 45°, and the knees were flexed up to 70°, with the feet kept free of the cast. Follow-up examinations were conducted every week or every 2 weeks on an outpatient basis. If the radiographs revealed that the callus was strong enough, the fracture was stabilized, and there was more callus at the fracture end, functional exercise was subsequently initiated. Normal walking and activities were gradually restored based on the healing of the fracture (2–3 months).

### Outcome evaluation

Based on the Knee Society Score method for evaluating treatment effects among children (7), the ratings were classified as follows: (1) excellent: the knee joint was fully extended, with 120° flexion, no pain, and shortening <1 cm; (2) good: fully extended, with 90° flexion, no or occasionally mild pain, slight angulation, and shortening <2 cm; (3) medium: extension difference of 10°, range of motion >60°, frequent mild pain, internal and external angle <10°, and shortening <3 cm; or (4) poor: extension difference of 10°, range of motion <60°, pain was obvious and long-lasting, the inner and outer angles were >10°, and shortening was >3 cm.

### Statistical analysis

Statistical analyses were conducted using SPSS 20.0 (IBM Corp., Armonk, NY, USA), and values are expressed as means ± standard deviations or numbers and percentages. Continuous variables were compared using the *t*-test, and the *χ*^2^ test was used for categorical variables. Statistical significance was set at *P* < 0.05.

## Results

From July 2012 to July 2018, a total of 18 out of 396 DDH patients aged 2–8 years with femur fractures after PFO were retrospectively reviewed. General data were retrieved from the hospital database, and clinical results were collected during follow-up. Meanwhile, patients with a follow-up time of <24 months or incomplete medical records were excluded from our study. From July 2012 to July 2018, a total of 396 children with DDH were treated at our institution, and 18 patients (3 patients were excluded because they fell from a height and because the exclusion did not differ by treatment) who presented with postoperative femur fractures were included in the study. Ten patients sustained fractures after plate removal: nine after a fall (from standing, twisting, or walking) and one spontaneously (without a fall). Moreover, eight patients experienced fractures early postoperatively after open reduction and PFO (after low-energy trauma such as ground-level falls).

### Fracture after plate removal (10 patients) and early failure after index surgery (8 patients)

Ten patients sustained fractures after plate removal within 2 months, and eight patients experienced fractures during the first 3 months after open reduction and PFO (all patients had transverse fractures). In children who underwent implant removal, all the fractures occurred at the osteotomy site (*n* = 10), whereas in those in whom the implant was not removed, the fractures occurred in the screw holes (*n* = 5) and in the distal femur (*n* = 3). The youngest child in our series was 2 years and 1 month old, and the oldest was 8 years old at the time of femoral fracture. There were 10 patients in the ESIN group (complete periprosthetic fractures in patients aged 5–8 years) and 8 patients in the spica casting group (minimally displaced incomplete fracture in patients aged 2–5 years).

### Demographic characteristics and operation data

As shown in Table 1, there were significant differences in age and fracture pattern (*P* < 0.001). Conversely, no statistically significant difference was found between the two groups concerning gender and side. Compared with that in the ESIN group (6.4 days), the average length of hospitalization was longer in the hip spica casting group (9.5 days, *P* < 0.001). The surgical time was shorter in the hip spica casting group (24.8 ± 4.5 min) than in the ESIN group (34.7 ± 5.1 min) (*P* < 0.05).

**Table 1:** Comparison of demographic characteristics, operation data, functional outcomes, and complications between the two groups.

Variable	Group A (*n* = 10)	Group B (*n* = 8)	*P*-value
*n*	10 (55.6)	8 (44.4)	
Mean age (range), years	6.3 ± 1.5	3.9 ± 1.2	<0.001
Displaced fracture, *n* (%)	9 (90.0)	1 (12.5)	<0.001
Gender, *n* (%)			
Male	6 (60.0)	5 (62.5)	
Female	4 (40.0)	3 (37.5)	
Side, *n* (%)			
Left	5 (50.0)	5 (62.5)	
Right	5 (50.0)	3 (37.5)	
Average length of hospitalization, days	6.4 ± 1.6	9.5 ± 1.9	<0.001
Time to union, months	4.7 ± 0.8	4.6 ± 0.9	0.868
Surgical time, min	34.7 ± 5.1	24.8 ± 4.5	0.018
Knee function (Knee Society Score) (7)			
Excellent	8 (80%)	7 (87.5)	0.652
Good	1 (10%)	1 (12.5)	
Fine	1 (10%)	0	
Poor	0	0	
Refracture	0	2	
Leg length discrepancy	1	1	
Infection	1	0	
Nail irritation	1	0	

### Complications and functional outcomes

In the ESIN group, one child had postoperative wound infection, which was resolved with appropriate antibiotic therapy. One child experienced skin irritation at the nail tail, with local skin swelling that disappeared after the intramedullary nail was removed. Two children in the spica casting group experienced refractures at the surgical site. In addition, one child in the ESIN group and one child in the spica casting group had elongated affected limbs (both <1.5 cm), which barely affected their limb function. During subsequent follow-up visits, the function of the affected limbs returned to normal (Table 1).

## Discussion

Larger neck–shaft angles or anteversion angles in the proximal femur are often found in children aged 5–11 years with DDH, especially in those with complete dislocation. Therefore, we often perform PFO to correct abnormal neck–shaft angles or anteversion angles. In the pediatric population, LCPs have been applied for femoral osteotomy fixation. Moreover, in our hospital, the plates are routinely removed after the osteotomy has healed according to radiographic evidence (2, 8). However, the incidence of implant-related femoral fractures has recently been reported to range from 0.3% to 3.6% (4, 9). In our hospital, the rate was 4.5% (18/396), and the high level of stress shielding in the proximal femur exerted by the implant could be the main reason.

Once postoperative femoral fractures in children with DDH occur, both closed reduction and ESIN or hip spica casting with 7 days of skin traction could ultimately yield good radiographic outcomes (10). However, several non-radiographic outcome measures associated with ESIN and hip spica casting differ. Proponents of ESIN believe that early mobility and the absence of a hip spica cast are less burdensome to children and their families (11). However, compared with hip spica casting, ESIN is also associated with an increased intraoperative burden, such as a longer duration of anesthesia and fluoroscopy (12). Although the postoperative management of femur fractures in the hips of children with DDH remains controversial, our study revealed that the results were generally good when patients were treated with ESIN or hip spica casting. However, it is recommended that children aged 5–8 years be treated with ESIN because the technique is minimally invasive and has a low complication rate. In addition, ESIN enables faster walking recovery, which prevents secondary displacement.

In our study, the location of the fracture site in the femur was related to the position of osteotomy, which can be explained as “stress shielding.” Therefore, the implants should not be removed until solid union is observed at the osteotomy site. On the other hand, PFO was associated with femur fractures because the blood supply was disrupted, which may affect callus formation during fracture healing and increase the risk of delayed union or non-union of fractures (13). We also suggest that children should be followed up closely after the hardware is removed and that high-intensity activity should not be permitted until moderate or extensive remodeling at the osteotomy site is confirmed. In our institution, we recommend that hip spica casting for 8 weeks be performed for non-displaced femoral fractures in children aged 2–5 years and that active non-weight-bearing exercises be performed for another 4 weeks. In addition, ESIN is a quick and minimally invasive technique for treating displaced femoral fractures in patients aged 5–8 years and prevents secondary displacement.

Nevertheless, the present study has several limitations. First, the number of cases of postoperative femoral fractures was small, which limits generalizability; thus, larger sample sizes will further validate our results, and a stratified comparison (undisplaced vs. displaced) will be performed in future research. Second, we cannot determine whether clinical factors other than those we recorded, such as the postoperative range of motion in the hip, are related to fractures after osteotomy. Finally, because of its retrospective nature and observational findings, discrepancies and confounders may exist in the characteristics of the patients between groups; thus, a prospective or multicenter study may be needed in the future.

## Conclusion

Our study revealed that the radiological outcomes were generally good after ESIN or hip spica casting. In our study, hip spica casting was suggested for non-displaced femoral fractures in patients aged 2–5 years. In addition, ESIN was a quick and minimally invasive technique for treating displaced femoral fractures in patients aged 5–8 years and prevented secondary displacement.

## Data Availability

The original contributions presented in the study are included in the article/Supplementary Material, further inquiries can be directed to the corresponding author.
